# An Investigation of the Complexity of Maillard Reaction Product Profiles from the Thermal Reaction of Amino Acids with Sucrose Using High Resolution Mass Spectrometry

**DOI:** 10.3390/foods3030461

**Published:** 2014-08-07

**Authors:** Agnieszka Golon, Christian Kropf, Inga Vockenroth, Nikolai Kuhnert

**Affiliations:** 1 School of Engineering and Science, Jacobs University Bremen, Campus Ring 1, 28759 Bremen, Germany; E-Mail: a.golon@jacobs-university.de; 2 Henkel AG & Co. KGaA, Henkelstr. 67, 40589 Düsseldorf, Germany; E-Mails: christian.kropf@henkel.com (C.K.); inga.vockenroth@henkel.com (I.V.)

**Keywords:** mass spectrometry, Maillard reaction, carbohydrates, amino acids, complex mixture

## Abstract

Thermal treatment of food changes its chemical composition drastically with the formation of “so-called” Maillard reaction products, being responsible for the sensory properties of food, along with detrimental and beneficial health effects. In this contribution, we will describe the reactivity of several amino acids, including arginine, lysine, aspartic acid, tyrosine, serine and cysteine, with carbohydrates. The analytical strategy employed involves high and ultra-high resolution mass spectrometry followed by chemometric-type data analysis. The different reactivity of amino acids towards carbohydrates has been observed with cysteine and serine, resulting in complex MS spectra with thousands of detectable reaction products. Several compounds have been tentatively identified, including caramelization reaction products, adducts of amino acids with carbohydrates, their dehydration and hydration products, disproportionation products and aromatic compounds based on molecular formula considerations.

## 1. Introduction

The chemical composition of the raw materials of food is rather well understood; its chemical composition changes, however, completely, upon processing. Most food consumed is processed prior to consumption by thermal treatment, including cooking, frying, roasting, baking and storage, including drying, pickling or fermentation [1]. Upon these processing steps, the original food constituents undergo remarkable chemical changes producing a myriad of novel compounds. For roasted coffee, only 50% of green bean components remain chemically unchanged, whereas the rest are being chemically modified, resulting in a material composed of an unknown structure, usually referred to as coffee melanoidins [2]; while for cocoa powder, around 75% of the material produced through food treatment is unidentified. The situation is similar for most other processed foods, and nowadays, the majority of chemical structures resulting from such a processing is yet to be identified. In 1912, Louis Camille Maillard, for the first time, discovered chemical changes during food processing, describing the reaction between amino acids and sugars at elevated temperatures, typical for food processing conditions [3]. Since then, only a few defined reaction products of food processing have been isolated and structurally characterized [4,5].

The Maillard reaction is one of the most important processes that takes place in food processing and storage. In the first step of the reaction, Amadori compounds are formed and considered as precursors of aroma, color and flavor. In the next steps, series of rearrangements, dehydrations and cyclizations occur to produce advanced glycation end (AGE) products. The Maillard reaction products (MRPs), especially Amadori compounds and melanoidins (high molecular weight compounds), are currently receiving a great deal of interest due to their reported health-promoting properties and their potentials as functional food ingredients [6]. Melanoidins, for example, represent an important part of human diet, with an average intake of several grams per day [7]. Many studies are focused on the high antioxidant capacity of MRPs in model systems and food materials, such as beer, coffee and bakery products [8,9]. Moreover, the antioxidants, antimicrobial and cytotoxic properties of MRPs have been reported [10,11,12].

The MRPs of cysteine are responsible for meat-like aromas, for example, 2-methyl-3-furanthiol, 2-furfurylthiol or 3-mercapto-2-butanone [8,13]; those of lysine and arginine have been detected in many bakery products, in the crust or crumb of bread, and in roasted coffee. Despite several investigations, the great complexity of Maillard reaction products is still a challenge for food chemists. Much attention has been given to the reactions of sugars, such as glucose and fructose and other monosaccharides, whereas food constituents important to browning include disaccharides, for example sucrose, extensively used in confectionery and pastry products [14,15].

To obtain a comprehensive picture of the Maillard chemistry underlying food processing, further investigations and the development of suitable analytical tools are necessary. Fourier Transform Ion Cyclotron mass spectrometry (FT ICR-MS) has been used only on a few occasions in the analysis of food materials. It has been successfully applied in our group to characterize the composition of black tea thearubigins, roasted coffee beans and the thermal decomposition products of starch [16,17,18,19].

The objective of this study is to better understand the composition of unresolved complex mixtures of food materials upon heating by employing the analytical technology and data interpretation approaches. The study focuses on the reaction products formed, when lysine (**1**), arginine (**2**), aspartic acid (**3**), tyrosine (**4**), serine (**5**) and cysteine (**6**) are heated with sucrose (**7**). High resolution mass spectrometry as a direct infusion was applied for all of the samples. Moreover, FT ICR-MS was used for the reactions of cysteine and serine with sucrose. Thus, generated molecular formula lists were subjected to graphical interpretation tools, such as the van Krevelen analysis, in order to provide more information about structural trends.

## 2. Experimental Section

### 2.1. Sample Preparation

All chemicals (analytical grade) were purchased from Sigma-Aldrich (Germany). All components of Maillard reaction mixtures were ground in a mortar, mixed together, dissolved in 1 mL of water and heated at 200 °C in the oven with a power of 1.2 kW. The heated samples were then stored at room temperature. Heated products were dissolved in methanol/water (1:1, v/v, 1 mL) and used for mass spectrometry analyses.

### 2.2. Methods

#### 2.2.1. ESI-TOF-MS

High-resolution mass spectra were recorded using a Bruker Daltonics micrOTOF instrument (Bruker Daltonics, Bremen, Germany) employing both negative and positive electrospray ionization modes. The micrOTOF Focus mass spectrometer (Bruker Daltonics) was fitted with an ESI source, and internal calibration was achieved with 10 mL of 0.1 M sodium formate solution. Calibration was carried out using the enhanced quadratic calibration mode. All MS measurements were performed in both negative and positive ion modes.

#### 2.2.2. FT ICR-MS

Ultra high resolution mass spectra were acquired using a Bruker (Bremen, Germany) solarix Fourier Transform Ion Cyclotron Resonance mass spectrometer (FTICR-MS) with a 12 T refrigerated superconducting cryo-magnet. The instrument was equipped with a dual electrospray ion source with ion funnel technology. The spectra of the samples were recorded in electrospray ionization positive and negative ion modes using direct infusion with a syringe pump with a flow rate of 120 μL/h.

## 3. Results and Discussion

### 3.1. Reactions of Amino Acids with Disaccharides

**Figure 1:**
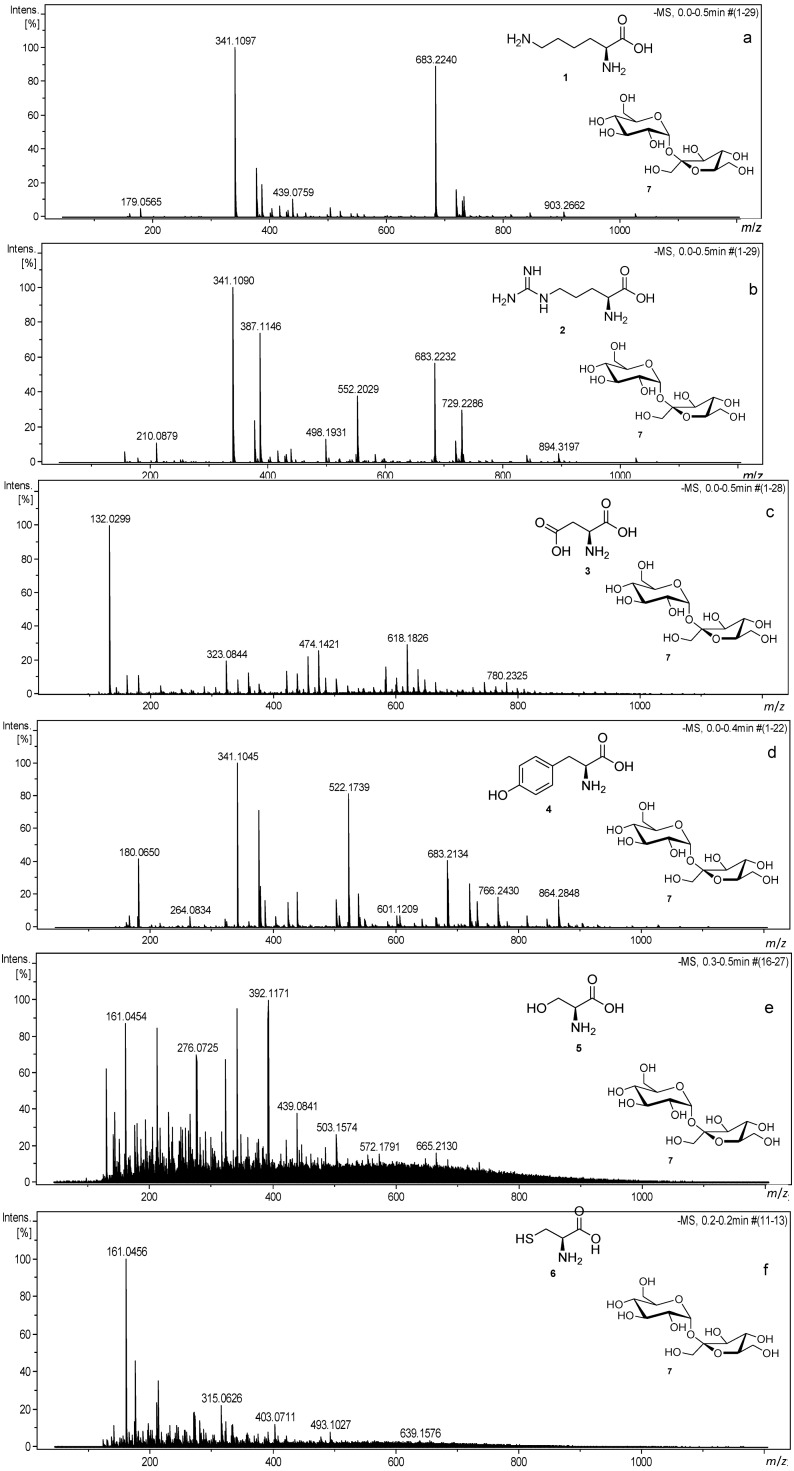
Mass spectra for heated: (**a**) lysine:sucrose (1:2), (**b**) arginine:sucrose (1:2), (**c**) aspartic acid:sucrose (1:2), (**d**) tyrosine:sucrose (1:2), (**e**) serine:sucrose (1:2) and (**f**) cysteine:sucrose (1:1) in negative ion mode using a direct infusion into an ESI-TOF-MS instrument.

We have chosen different amino acids, namely arginine, lysine, aspartic acid, tyrosine, serine and cysteine, to probe their reactivity with disaccharides under thermal treatment. As disaccharides, the two most common and relevant derivatives in food, sucrose and lactose, were chosen. The mixtures of two selected disaccharides with selected amino acids were heated at various temperatures ranging from 150 to 200 °C. Heating parameters were optimized based on color formation and the recorded mass spectra of products heated at different temperatures. Experiments performed at different heating conditions showed that around a half an hour reaction time at 200 °C are the most suitable parameters to obtain the desired brown material with in excess of 90% of the starting materials being consumed in the reaction. Carbohydrates were mixed with amino acids with different proportions (1:1, 2:1 and 1:2, v/v), which correspond to their composition in typical food products. The reactions were performed with sucrose and lactose, although in this contribution, we focus mainly on the reactions with sucrose. The significant difference between two carbohydrates was observed, and lactose in contrast to saccharose in the reactions with amino acids resulted in more complex mass spectra as judged by the number of resolved peaks observed. This finding might be explained by the stereochemistry of lactose having the axial C4-OH substituent of galactose. Upon heating, this can cause a higher level of dehydration and, consequently, an increased number of intermediates. Mass spectra for heated mixtures of amino acids with sucrose are displayed in Figure 1. All of the amino acids exhibit different reactivities. Mass spectra from the reactions of sugars with lysine and tyrosine are relatively simple, but more complex for arginine and extremely complex for cysteine and serine. The term simple hereby signifies the observation of a small number of intense observed signals (≤100), whereas the term complex refers to a large number of signals (≥400) usually at lower intensities. The complexity can be easily visualized by the number of signals with an S/N ratio higher than 10 in MS spectra, and the number of signals for the reactions are: 221-Lys:Suc (1:1), 495-Cys:Suc (1:1) and 691-Ser:Suc (1:2) (Table 1).

**Table 1:** The number of peaks observed for samples in ESI-TOF-MS and FT ICR-MS in negative ion mode.

No.	Sample	Number of Signals with S/N > 10	Number of Signals with S/N > 3
1	Arginine-sucrose ^a^	126	714
2	Lysine-sucrose ^a^	221	670
3	Aspartic acid-sucrose ^a^	297	796
4	Tyrosine-sucrose ^a^	420	916
5	Serine-sucrose ^a^	691	885
6	Cysteine-sucrose ^a^	495	791
7	Serine-sucrose ^b^	2682	4698
8	Cysteine-sucrose ^b^	5193	8527

^a^ ESI-TOF-MS; ^b^ FT ICR-MS.

The examination of the spectra aimed initially at the identification of typical caramelization reactions products [20,21]. From the experimental mass lists, molecular formula lists were generated and compared to those obtained for typical caramelization reactions in the absence of amino acids. The comparison of peaks with identical accurate mass values and, therefore, molecular formulae, if compared to previous studies on caramelization processes, allowed the tentative identification of around 70 caramelization products, including oligomers of hexoses, with up to a maximum of six monomeric units, both dehydration products of monomeric hexoses and oligomeric hexoses showing successive a loss of up to seven water molecules, depending on the number of monomers. Hydration products with up to two water molecules added to an oligomeric carbohydrate were detected [19,20]. Fragmentation products after the redox disproportionation reaction were found and also aromatic compounds after excessive dehydration. The reaction products between amino acids and sucrose gave oligomers of amino acid conjugated to hexoses after the hydrolysis of sucrose in the aqueous reaction medium (either fructose or glucose; MS*^n^* data revealed exclusively neutral losses for hexose fragments and did not allow distinction between fructose and glucose) with a maximum of four carbohydrate units, the dehydration products loosing up to six water molecules and hydration products with up to two water molecules added to the oligomeric products. In Table 2, a mass list of some of the compounds for one of the studied mixtures, the reaction between aspartic acid with sucrose, is shown. In this case, the reaction products of aspartic acid with sucrose with a maximum of four carbohydrate units, their dehydration products loosing up to four water molecules and hydration products with up to one water molecule were found. In most of the cases, signals corresponding to the molecular formulae of dehydration products of amino acid conjugated to carbohydrates with the loss of two water molecules appeared to have the highest intensities.

The studies on processed food components by many research groups are focused on single compounds, such as 5-HMF, acrylamide or heteroaromatic cyclic amines (MeIQ, PhIP) formed during cooking or baking [22]. Our work presented here allows a more global view on the many products so far neglected in thermal processing. It clearly shows that many additional compounds of related chemical structures are present in processed food, with a crude and tentative structure classification based on molecular formulae considerations possible.

**Table 2:** ESI-TOF-MS data in negative ion mode of a reaction of aspartic acid with sucrose.

No.	Assignment	Molecular Formula	Theoretical *m*/*z* [M − H]^−^	Experimental *m*/*z* [M − H]^−^	Error (ppm)
1	Asp-Glu/Fru	C_10_H_17_NO_9_	294.0831	294.0831	−0.1
2	Asp-Glu/Fru + H_2_O	C_10_H_19_NO_10_	312.0936	312.0935	0.2
3	Asp-Glu/Fru-2 × H_2_O	C_16_H_23_NO_12_	420.1147	420.1167	−4.6
4	Asp-Glu/Fru-H_2_O	C_16_H_25_NO_13_	438.1253	438.1236	3.9
5	Asp-Suc	C_16_H_27_NO_14_	456.1359	456.1357	0.3
6	Asp-Suc + H_2_O	C_16_H_29_NO_15_	474.1464	474.1472	−1.6
7	Asp-3 × Glu/Fru-3 × H_2_O	C_22_H_31_NO_16_	564.1570	564.1583	−2.3
8	Asp-3 × Glu/Fru-2 × H_2_O	C_22_H_33_NO_17_	582.1676	582.1680	−0.7
9	Asp-3 × Glu/Fru-H_2_O	C_22_H_35_NO_18_	600.1781	600.1789	−1.3
10	Asp-3 × Glu/Fru	C_22_H_37_NO_19_	618.1887	618.1879	1.4
11	Asp-3 × Glu/Fru + H_2_O	C_22_H_39_NO_20_	636.1993	636.1990	0.4
12	Asp-4 × Glu/Fru-4 × H_2_O	C_28_H_39_NO_20_	708.1993	708.2048	−7.7
13	Asp-4 × Glu/Fru-3 × H_2_O	C_28_H_41_NO_21_	726.2098	726.2139	−5.5
14	Asp-4 × Glu/Fru-2 × H_2_O	C_28_H_43_NO_22_	744.2204	744.2236	−4.3
15	Asp-4 × Glu/Fru-H_2_O	C_28_H_45_NO_23_	762.2310	762.2303	0.8
16	Asp-4 × Glu/Fru	C_28_H_47_NO_24_	780.2415	780.2385	3.9
17	Asp-4 × Glu/Fru + H_2_O	C_28_H_49_NO_25_	798.2521	798.2507	1.7

### 3.2. FT ICR-MS Measurement of Maillard Reactions of Serine and Cysteine with Sucrose

**Figure 2:**
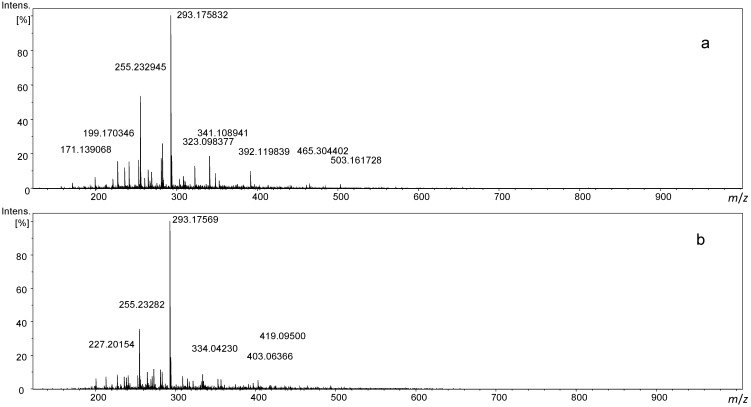
Direct infusion ESI-FT-ICR mass spectrum of a reaction of (**a**) sucrose with serine and (**b**) sucrose with cysteine in negative ion mode.

The reactions of serine and cysteine with sucrose resulted in extremely complex MS spectra with signal numbers exceeding several thousand and were therefore analyzed by FT ICR-MS, providing ultimate resolution. The samples were measured as a direct infusion in aqueous methanol solution using a 12T FT ICR-MS instrument with ESI ionization in both positive and negative ion mode. The data obtained provides a comprehensive overview of all products containing the reaction products detectable by ESI-MS. Representative mass spectra are shown in Figure 2. Further expanded mass spectra are available in the Supplementary Materials (Figures S1–S3).

From the data, mass lists were generated with an S/N ratio above four and a relative intensity higher than or equal to 0.1%. A detailed discussion on the cut-off level of the signal intensity and the distinction between noise and signals corresponding to real food processing products has been given in earlier references [1,16]. In the case of serine, 5340 signals were present, while for cysteine 8530. This confirms that the presence of sulfur in the molecule leads to the formation of several new MRPs. It is worth mentioning that the number of detected ions is the minimum number of compounds present in the sample, which must be multiplied by the number of potential isomers, consequently leading to thousands of compounds.

From the mass lists, molecular formula lists were generated, accepting an error below 1 ppm and the presence of C, H, O and N for serine and C, H, O, N and S for cysteine for all signals with a relative intensity of 0.1% of the base peak. The data were then subjected to interpretation tools.

Molecular formulae have been assigned for almost 600 of the most intense peaks for the reactions with serine and cysteine. The majority of assigned signals contain nitrogen atoms. In the case of cysteine, around 90 molecular formulae contain one or more sulfur atoms, for example an adduct at *m/z* 264.054607 with the molecular formula C_9_H_15_NO_6_S, which corresponds formally to the reaction products between cysteine and hexose after dehydration, or at *m*/*z* 282.065162 with the molecular formula C_9_H_17_NO_7_S, derived from the reaction between cysteine and hexose.

### 3.3. Graphical Data Interpretation

**Figure 3:**
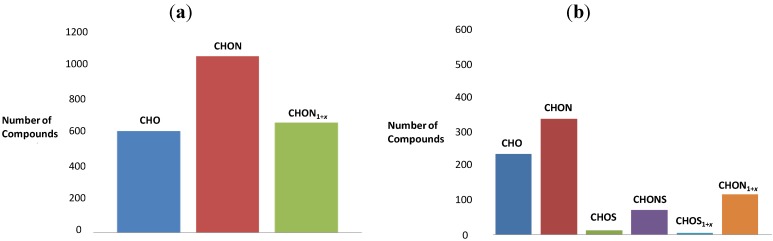
The number of compounds with the elemental composition (**a**) CHO, CHON and CHON_1+*x*_ for the reaction of serine with sucrose and (**b**) the elemental composition CHO, CHON, CHOS, CHONS, CHOS_1+*x*_ and CHON_1+*x*_ for the reaction of cysteine with sucrose, analyzed by negative ion mode FT ICR-MS.

**Figure 4:**
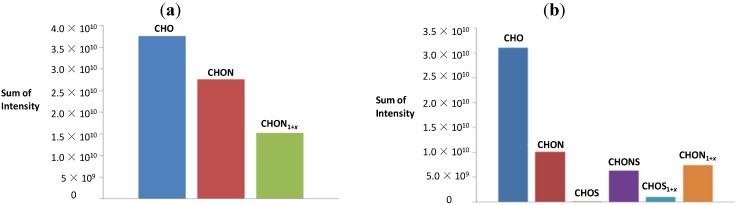
The sum of intensities for the compounds with the elemental composition (**a**) CHO, CHON and CHON_1+*x*_ for the reaction of serine with sucrose and (**b**) the elemental composition CHO, CHON, CHOS, CHONS, CHOS_1+*x*_ and CHON_1+*x*_ for the reaction of cysteine with sucrose analyzed by negative ion mode FT ICR-MS.

To visualize differences in product formation, we generated graphs showing a number of compounds and a second graph displaying the sum of ion intensities summed up over all signals with the respective elemental composition, CHO, CHON and CHON_1+*x*_ for the reaction of serine with sucrose and CHO, CHON, CHOS, CHONS, CHOS_1+*x*_ and CHON_1+*x*_ for the reaction of cysteine with sucrose (Figure 3). Each column represents the number of compounds with a defined elemental composition. For serine, the number of nitrogen-containing molecules is higher than nitrogen-free molecules, and more than a half of the latter contains more than one nitrogen atom; although the total intensities of CHO molecules are higher than for nitrogen-containing molecules (Figure 4). The sum of intensities of pseudomolecular ions corresponding to molecular formulae with one nitrogen atom are higher than for the molecules containing two and more nitrogen atoms. For cysteine, the number of compounds with the CHON elemental composition is higher than for CHO. The number of molecules with nitrogen and sulfur atoms is lower than that of CHON molecules. The highest intensities correspond to CHO molecules, followed by CHON, CHON_1+*x*_, CHONS, CHOS_1+*x*_ and CHOS. The sum of intensities of molecules with one nitrogen atom are slightly higher than for ones with more than one nitrogen atom (Figure 4).

### 3.4. The van Krevelen Diagrams for the Reaction of Serine with Sucrose

The van Krevelen diagrams were generated from the molecular formulae lists. In this diagram, elemental ratios, such as H/C or O/C, were calculated from the molecular formulas and plotted in two-dimensional graphs, with every point on the graph corresponding to one analyte with a defined elemental composition in the sample. These diagrams allow the tentative classification of classes of compounds based on their characteristic elemental ratio boundaries and the identification of possible reaction trends [1,23].

**Figure 5:**
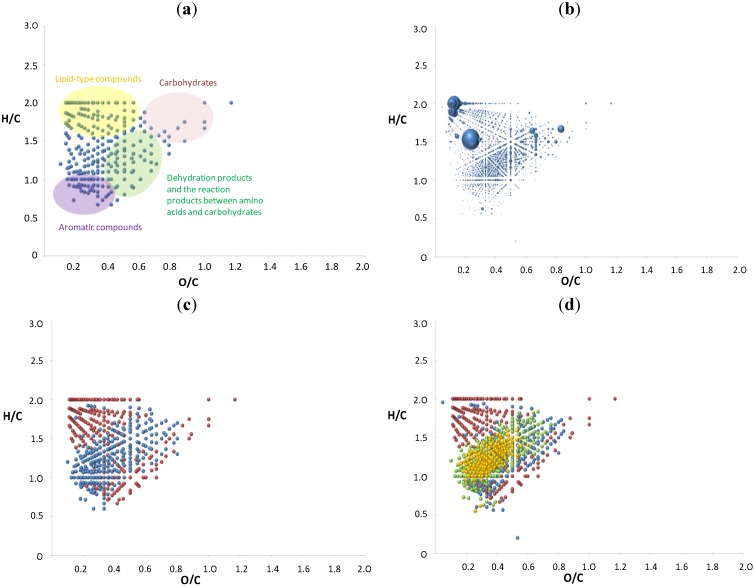
The van Krevelen diagrams of the reaction of sucrose with serine: (**a**) with colored groups of compounds; (**b**) with the intensities; (**c**) with nitrogen-free (red points) and nitrogen-containing compounds (blue points); (**d**) with nitrogen-free (red points) compounds and one nitrogen atom (blue points), two (green), three (yellow) and four (violet) nitrogen atoms in negative ion mode.

The van Krevelen diagrams were generated in several variations, with one plot showing all analytes, one bubble plot showing intensity coded data points and two-three plots showing Maillard products with color coding according to different elemental compositions. The van Krevelen diagram of the reaction of sucrose with serine is shown in Figure 5a. In typical elemental ratio boundaries for carbohydrates (right top corner), products can be observed; in the middle of the graph are reaction products between amino acid and carbohydrate and carbohydrate after dehydration. Many compounds after gradual dehydration lay on the diagonal of the plot towards its origin. The second graph shows the same points with their intensities (Figure 5b), and thus, the three compounds with the highest intensities of ions observed correspond to two lipid-type compounds and one carbohydrate. The observation of ions with a lipid-type elemental ratio comes as a surprise, and we cannot exclude an erroneous molecular formulae assignment or other measurement artefact at this point. The reaction products between amino acid and sugar are created in moderate intensities. Moreover, many homologous series can be observed in the graph, such as dehydration products of amino acid-sugar reaction products. In Figure 5c, red points represent nitrogen-free compounds, while blue nitrogen-containing compounds. Within the mixture, 45% of the ions are CHO compounds free of nitrogen and, therefore, originate from caramelization reactions, whereas the remaining 55% contain nitrogen and must be considered as real Maillard products. The data points, which belong to carbohydrates, can be easily seen by following dehydration products on the line with the negative slope toward the origin in the middle of the graph. The next group of nitrogen-free compounds appear in the range of 1.5–2.0 for the H/C ratio and 0.0–0.6 for the O/C ratio, corresponding to disproportionation reaction products. The nitrogen-containing products are located mainly in the middle of the graph and belong to the reaction products between serine and sucrose. Figure 5d displays the distribution of nitrogen-containing compounds with respect to the nitrogen numbers in molecules. The red points correspond to nitrogen-free compounds, while the blue points are the molecules with one nitrogen atom, green with two and yellow with three nitrogen atoms. The distribution shows that one-nitrogen-containing molecules are placed next to the carbohydrate-type molecules on the diagonal line towards point zero, suggesting the dehydration products. From these data, it must be concluded that oligopeptide-like structures are formed with three and more nitrogen atoms, with oligomerization playing an important role in the Maillard reaction, as shown earlier for caramelization [19,20,21] Compounds with two nitrogen atoms are mainly around 0.5 and 1.5 for the O/C and H/C ratio, respectively, and go toward the more aromatic region. On the other hand, compounds with three nitrogen atoms are placed mainly around the point of 0.3 for O/C and 1.2 for H/C, forming one circle.

### 3.5. The van Krevelen Diagrams for the Reaction of Cysteine with Sucrose

**Figure 6:**
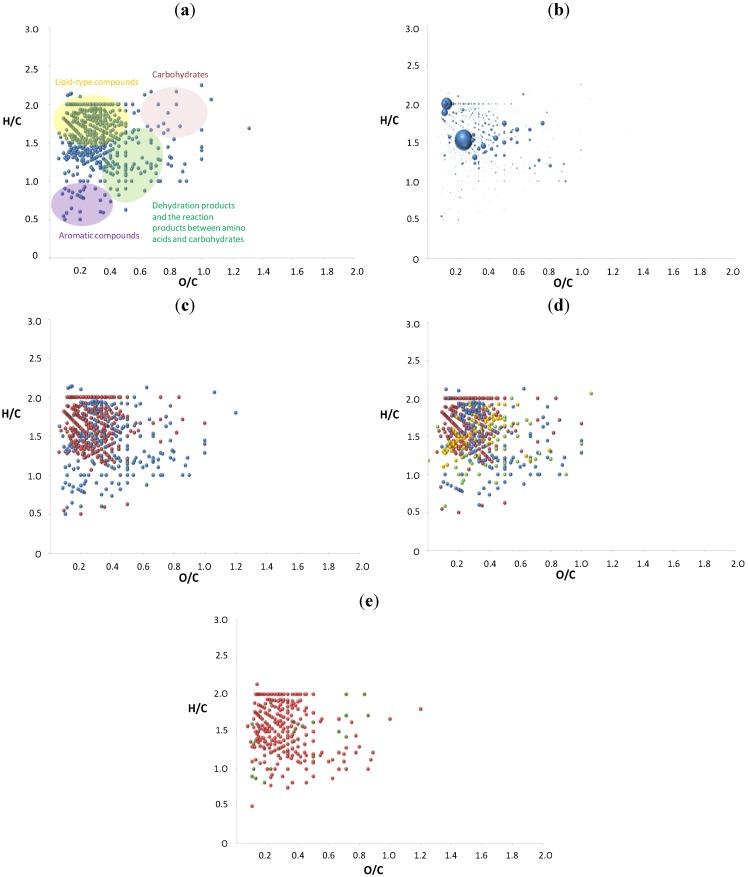
The van Krevelen diagrams of the reaction of sucrose with cysteine: (**a**) with colored groups of compounds; (**b**) with the intensities; (**c**) with nitrogen-free (red points) and nitrogen-containing compounds (blue points); (**d**) with nitrogen-free (red points) compounds and one nitrogen atom (blue points), two (green), three (yellow) and four (violet) nitrogen atoms; (**e**) with sulfur-free (red points) and sulfur-containing (green points) compounds in negative ion mode.

The profile of the van Krevelen diagram for the reaction of cysteine with sucrose is similar to the one with serine and saccharose, although the number of points in the aromatic region is higher (Figure 6a). The intensities of carbohydrate-type compounds are not as high as in the case of serine, while the signals of some of the reaction products between the amino acid and carbohydrate appear with relatively high intensities (Figure 6b). This can suggest the intensive conversion of sucrose into various products. In Figure 6c, with red nitrogen-free and blue nitrogen-containing compounds, most of the nitrogen-free compounds appearing in the left upper part are characteristic of lipid-type compounds. Some of them are seen in the aromatic region after the successive dehydration reaction. The nitrogen-containing compounds are almost evenly spread over the whole graph. In Figure 6d, we can observe the distribution of nitrogen-containing compounds. The N-containing compounds show a pattern resembling the van Krevelen plot of serine-derived Maillard products. As for serine, the products with one nitrogen atom are distributed in the whole diagram and are placed in the upper left side, in the aromatic region and in the middle of the graph. The last corresponds to the reaction products between cysteine and carbohydrate. The products with three nitrogen atoms are located mainly around the point of 0.2 for O/C and 1.5 for H/C, which is in agreement with the reaction of serine. In the same region, most of the compounds with four nitrogen atoms are observed. In Figure 6e, the allocation of sulfur-containing molecules is shown. Most of these molecules are located in the middle of the graph, being the reaction products between cysteine and sucrose. Some of them are placed in the aromatic region with a low O/C ratio after successive dehydration.

## 4. Conclusions

To conclude, we have characterized complex mixtures formed upon the heating of different amino acids with sucrose using powerful mass spectrometry techniques. We have shown that thermal treatment of reactive amino acids with carbohydrates results in several thousand reaction products. The different reactivity of free amino acids has been demonstrated. In particular, lysine and tyrosine in the presence of sucrose yielded the simple spectra, followed by arginine and aspartic acid, while cysteine and serine produced the highest number of compounds. We have identified the whole range of caramelization products, including oligomers of carbohydrates with up to six carbohydrate units, dehydration products of oligomers loosing up to a maximum of seven water molecules, hydration products of sugar oligomers, disproportionation products and aromatic compounds. Except caramelization reaction products, many reaction products between amino acids and carbohydrates have been found in the samples, including adducts of amino acids with carbohydrates up to four carbohydrate units. The formation of the reaction products between amino acids and sucrose without two water molecules was favored. The compounds with up to four nitrogen atoms incorporated in the structures have been formed and illustrated in the graphs. Several sulfur-containing MRPs have been identified in the reaction of sucrose with cysteine (e.g., the addition products of cysteine and hexose). Because of the high accuracy of FT ICR-MS, the formula assignment for nitrogen- and sulfur-containing molecules was successful. The van Krevelen diagrams generated form FT ICR-MS data turned out to be useful tools in complex mixture characterization. The work described herein provides a comprehensive overview on Maillard chemistry, without which our life would be tasteless.

## Supplementary Materials

Supplementary File 1
