# Supplementary material for: An Investigation of the Complexity of Maillard Reaction Product Profiles from the Thermal Reaction of Amino Acids with Sucrose Using High Resolution Mass Spectrometry
Source: Foods. 2014 Aug 7;3(3):461–75. doi: 10.3390/foods3030461 (PMC5302257; doi:10.3390/foods3030461)
Supplement: Supplementary File 1 [file foods-03-00461-s001.pdf]

Supplementary Materials: An Investigation of the Complexity of Maillard Reaction Product Profiles from the Thermal Reaction of Amino Acids with Sucrose Using High Resolution Mass Spectrometry

Agnieszka Golon, Christian Kropf, Inga Vockenroth and Nikolai Kuhnert

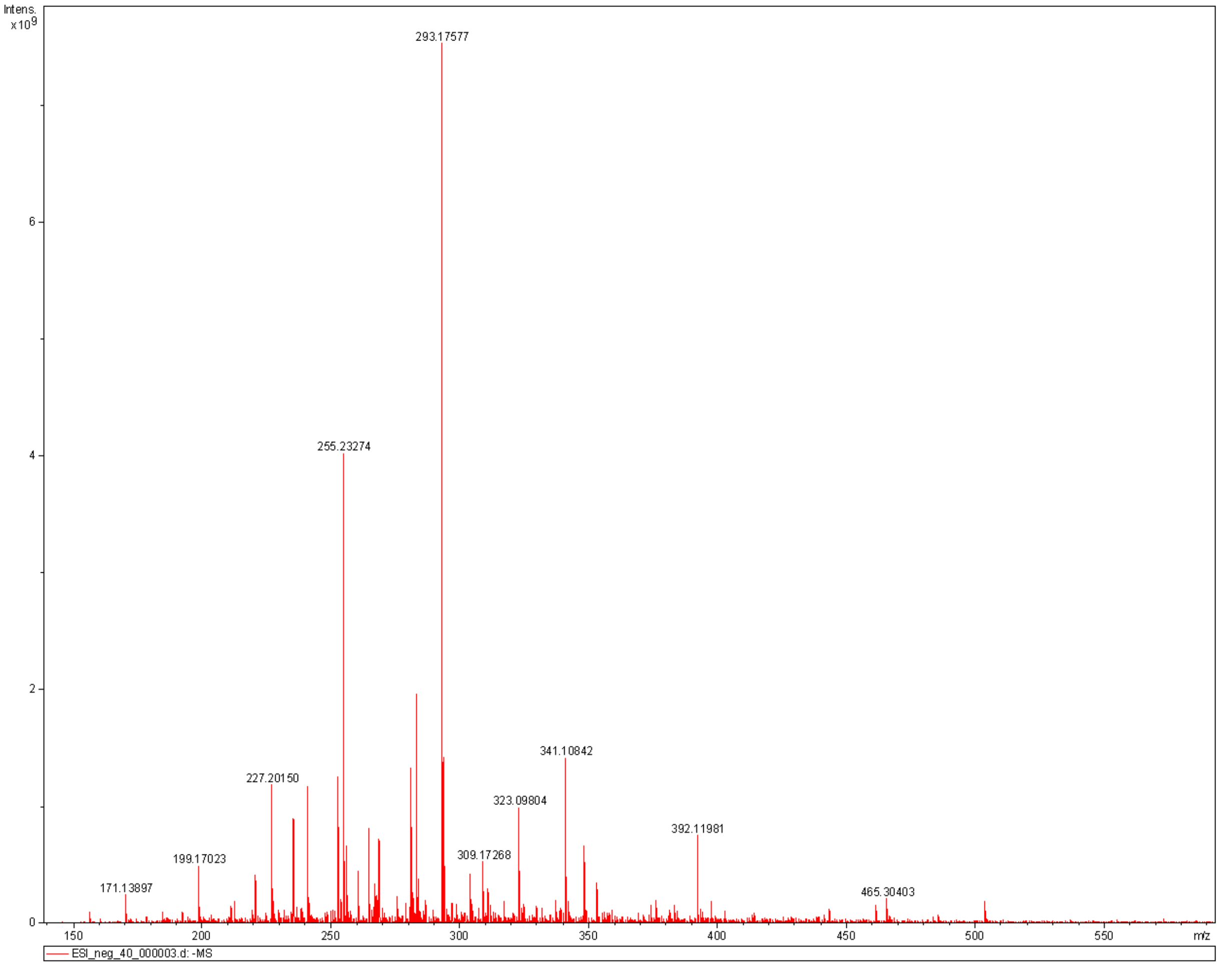

Figure S1. Full ESI-FI-ICR-MS of sucrose reacted with serine in negative ion mode.

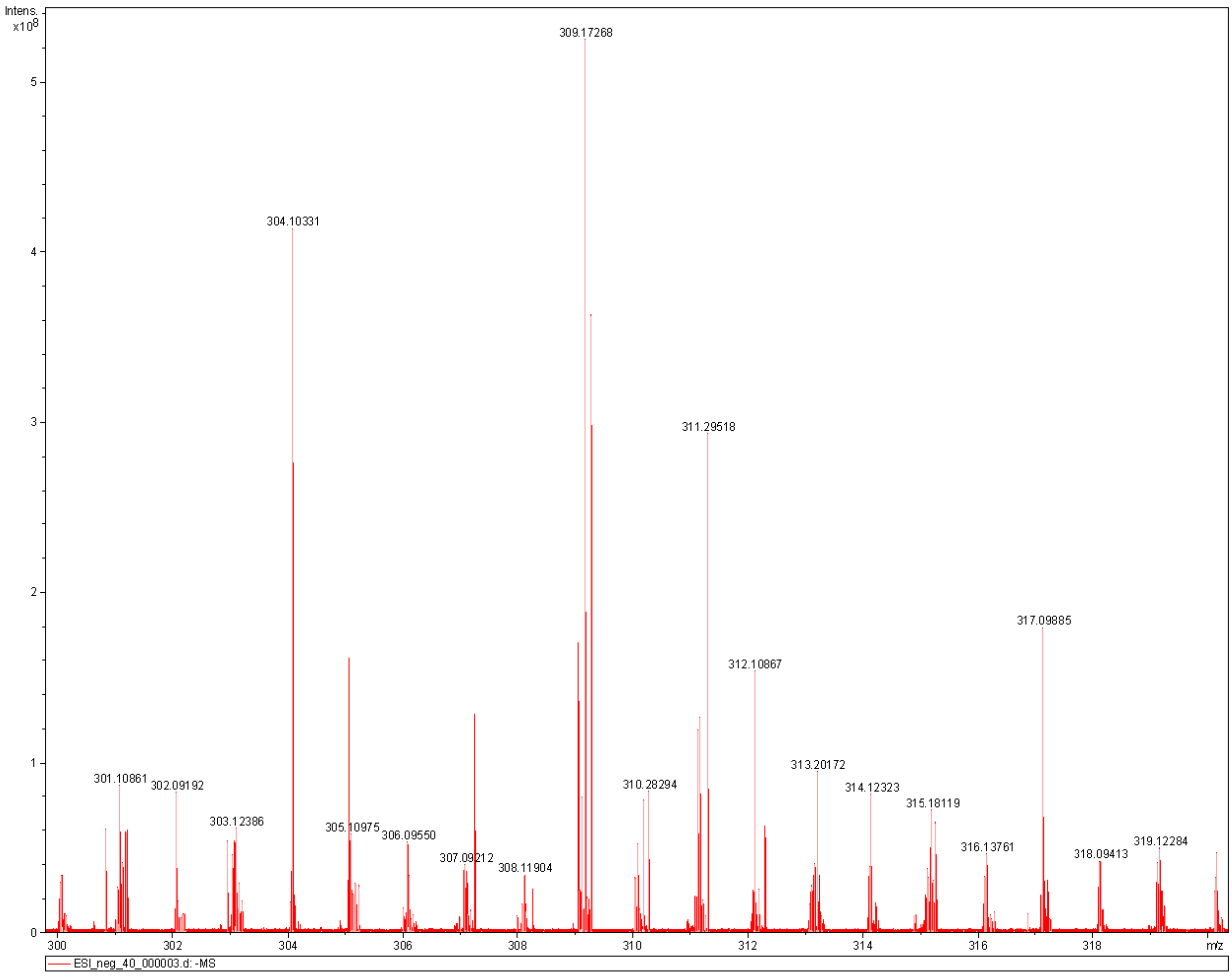

Figure S2. Expanded region (*m*/*z* 300–320) of ESI-FI-ICR-MS of sucrose reacted with serine in negative ion mode.

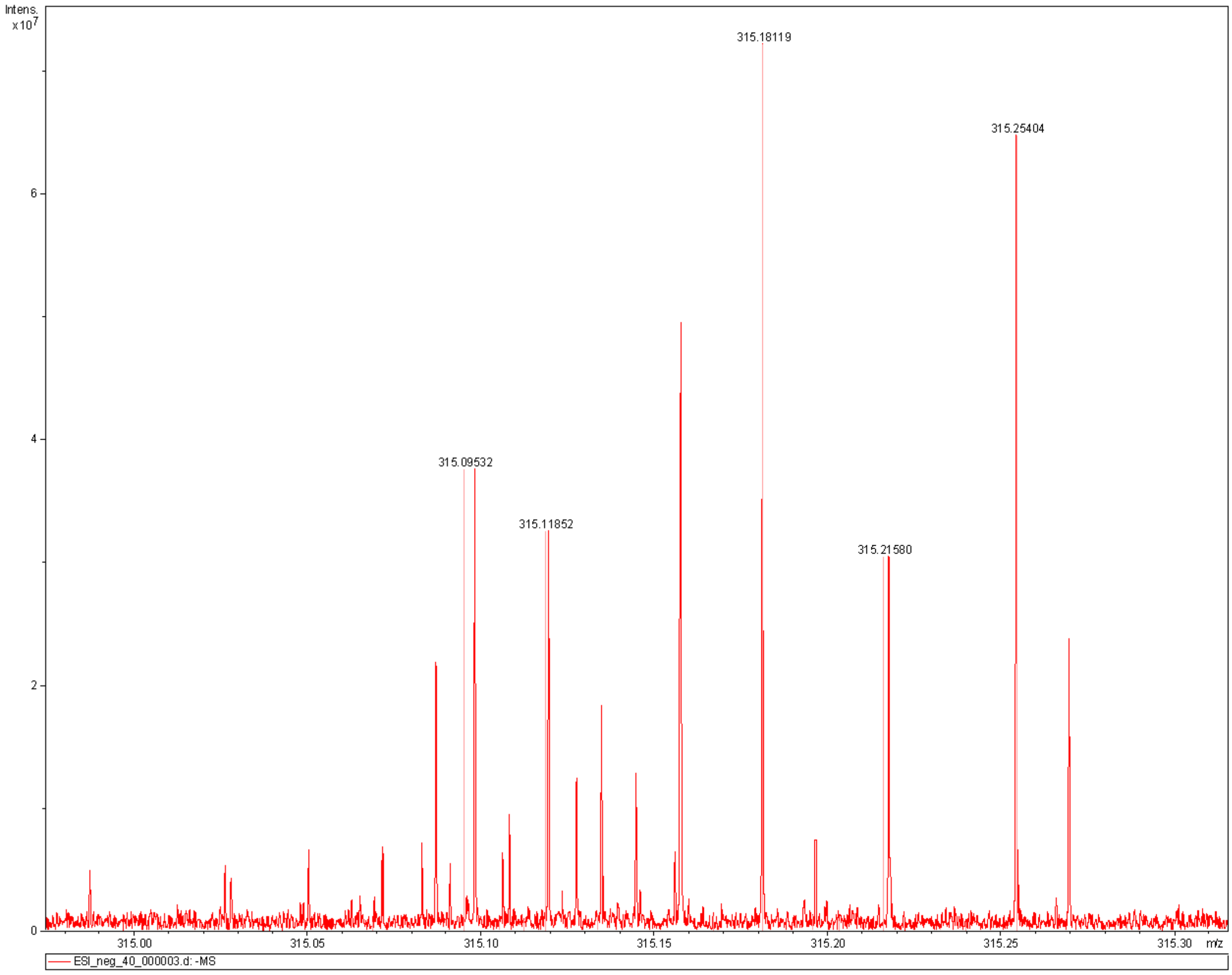

Figure S3. Expanded region (*m*/*z* 316–318) of ESI-FT-ICR-MS of sucrose reacted with serine in negative ion mode.

© 2017 by the authors; licensee MDPI, Basel, Switzerland. This article is an open access article distributed under the terms and conditions of the Creative Commons by Attribution (CC BY) license (http://creativecommons.org/licenses/by/4.0/).
